# Randomized Crossover Study Showing Nurse-Led Same Day Review Replacing Next Day Review in Uneventful Phacoemulsification to Be Safe and Efficacious

**DOI:** 10.1155/2017/1261698

**Published:** 2017-03-23

**Authors:** Jennifer W. H. Shum, Janice J. C. Cheung, Monica M. N. Lee, Oscar G. W. Wong, Kenneth K. W. Li

**Affiliations:** ^1^Department of Ophthalmology, The University of Hong Kong, Pok Fu Lam, Hong Kong; ^2^Department of Ophthalmology, United Christian Hospital, Kwun Tong, Hong Kong; ^3^Department of Pathology, The University of Hong Kong, Pok Fu Lam, Hong Kong

## Abstract

*Purpose*. To study whether nurse led same-day review (SDR) after uneventful phacoemulsification can replace next-day review (NDR) in terms of safety and efficacy. *Setting*. Patients are recruited from an ophthalmology outpatient clinic in Hong Kong. *Design*. A prospective, randomized crossover study conducted from November 2012 to 2014. *Methods*. Inclusion criteria include cataract surgery naïve patients undergoing phacoemulsification under local anaesthesia. All patients were seen by our ophthalmic nurse 2 hours after surgery. Before undergoing phacoemulsification of the first eye, patients were randomized to be reviewed on day 1 or 7 after surgery. Surgeons and reviewing doctors were blinded to patient allocation. For the patients' second eye surgery, group allocation will cross over. Primary outcome measures include visual improvement and patient satisfaction questionnaire. Other measures include cataract characteristics, surgical details, and complications. Statistical tests include paired *t*-test, Wilcoxon signed rank test, and Chi-square test. *Results*. 164 eyes from 82 patients were available. Visual improvement, satisfaction, and complications were comparable between both groups. *Conclusions*. A nurse led SDR can replace NDR in uneventful phacoemulsification in terms of safety and efficacy. Patient satisfaction is also comparable in the setting of Asian culture and when transportation is not a major concern.

## 1. Background

Next day review (NDR) following phacoemulsification is actually more of a convention and tradition rather than being evidence based. The Cataract Surgery Guidelines published by the Royal College of Ophthalmologists (RCOph) in 1995 had recommended postoperative review within 48 hours after surgery. This recommendation was based on data from surveying postoperative complications at a time when the extracapsular cataract extraction (ECCE) technique was mainstream and wound integrity or prolapsed irises were major concerns. The National Cataract Survey conducted in 1993 showed the 3 most frequently occurring complications: corneal edema, raised intraocular pressure (IOP), and wound leak [1]. The most recent National Cataract Survey in 1997 collected data from surgeries, which comprised 80% of phacoemulsification and 20% of ECCE, showing a shift of the 3 most frequently occurring complications to corneal edema, raised IOP, and uveitis, [2] shifting the emphasis from wound-related complications to IOP spikes. The most recent Cataract Surgery Guidelines published by RCOph in 2010 states that NDR is no longer in widespread use, with many departments having replaced patient visit with a telephone call by a trained nurse [3]. The intervention rate in routine clinical review following uneventful phacoemulsification has also found to be low (2.8%), with the majority of these are avoidable or trivial [4].

As evidenced above, NDR following uneventful cataract surgery may not be an efficacious practice. It causes inconvenience and leads to additional cost to patients, most of which are elderly. It also casts a burden on health care cost and consumes clinic time, as one uncomplicated case of cataract generates approximately 5–8 outpatient appointments from surgery listing to closing case. However, the practice of NDR is still very common across Asia [5, 6].

The purpose of postoperative follow-up is multifold and includes the following: screen for complications, allow for intervention where necessary, allow the surgeon to review his surgical outcome which encourages surgical development, and assess and assure the patient. We propose that same day review (SDR) can replace traditional NDR in meeting the above goals, with other additional advantages. SDR is more effective in detecting postoperative IOP spike, as it has been found that IOP peaks at 3 to 7 hours postoperatively [7]. This is all the more important in glaucoma patients, as 19% of glaucoma patients were found to have peak IOP above 40 mmHg, as compared to 4% of patients without glaucoma. SDR is also more convenient for patients: it saves travel time and trouble, it allows immediate eye pad removal and immediate usage of postoperative eye drops, and it also allows earlier reassurance.

In this study, we will compare the visual outcome in patients undergoing SDR versus NDR and also patient satisfaction level in an urban and metropolitan setting, where travel time is less of an issue.

## 2. Methods

This is a prospective, randomized crossover study conducted from November 2012 to November 2014. Patients are recruited from the outpatient clinic at a tertiary eye centre in the eastern part of the Kowloon Peninsula of Hong Kong serving a population of around 1 million.

The study was approved by the Institutional Review Board of the Kowloon East Cluster Research Ethics Committee in Hong Kong (reference number KC/KE-12-0101/ER-2) and adhered to the tenets of the Declaration of Helsinki. Written informed consent was obtained. Indications for cataract surgery include visual acuity less than 0.5 together with a patient's wish for surgery. Inclusion criteria include patients with bilateral cataract that required surgery, willingness to undergo sequential phacoemulsification under local anaesthesia within 3 months, and age of at least 18. Exclusion criteria include only eye patients, end-stage glaucoma patients, minors, any intraoperative complications, conversion into ECCE and surgery done under general anaesthesia, illiteracy, and inability to complete a questionnaire.

**Figure 1:**
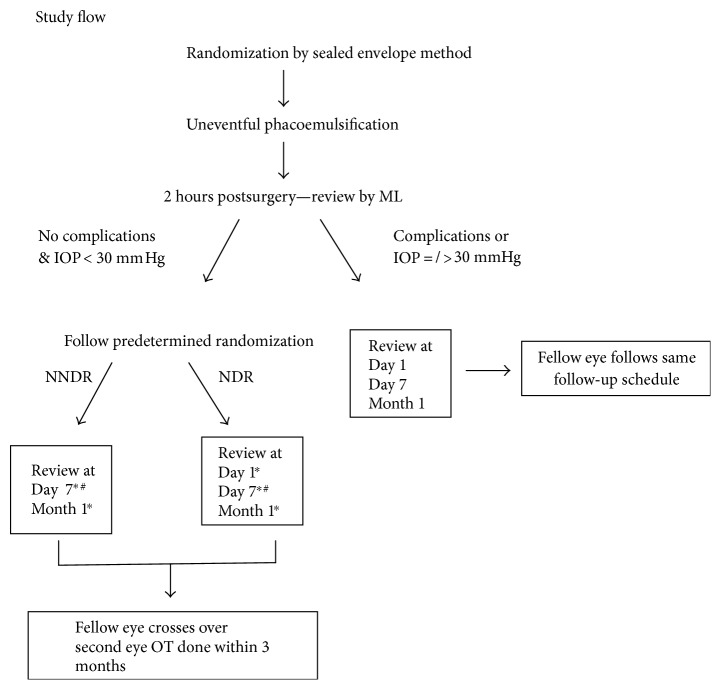
The outline of the study design. NDR: next day review; NNDR: no next day review. ^∗^Investigator will be blinded to patient allocation as he will examine the patient first before reviewing past clinical notes. ^#^Satisfaction questionnaire completed.

Before undergoing phacoemulsification of the first eye, randomized allocation was done via sealed envelope method. An outline of our study design is shown in Figure 1. Surgeons performing the surgery were blinded to group allocation. Doctors reviewing the patients' postoperation on either day 1 or day 7 were also blinded to group allocation, as they would examine the patient first before reviewing the patients' clinical records.

All patients were reviewed 2 hours postsurgery at our Day Surgery Center by an ophthalmic specialist nurse (ML) who had undergone accredited training in slit lamp examination. The timing of review at 2 hours after surgery was chosen as it helps identify those with a rising IOP and it gives consideration to patient convenience [7, 8]. The ophthalmology nurse specialist would perform the following: remove eye dressing, slit lamp examination to inspect for a round pupil configuration and intraocular lens centricity, check wound integrity via Seidel test, and IOP measurement via noncontact tonometry. Noncontact tonometry was chosen as the method for IOP measurement as it is less invasive, quick, convenient, and shown to agree well with Goldmann applanation [9]. The nurse specialist would then pad the patient's eye with maxitrol ointment (Alcon) and instruct the patient on postoperative care. The importance of symptoms such as severe ocular pain and reduced vision were explained, and contact numbers in case of emergency were given.

If no complications were seen and the IOP was lower than 30 mmHg, the patient would be told to start postoperative eye drops and followed up according to the next day review (NDR) or no next day review (NNDR) group schedule as allocated. NDR patients would be seen on 1 day, 7 days, and 1 month postsurgery. NNDR patients would be told to take off their eye dressing 4 hours after surgery and start postoperation eye drops, and they would be seen on 7 days and 1 month postsurgery. For these reviews, the patients would be seen at our outpatient clinic. The fellow eye of all patients will automatically crossover to the alternative group, with cataract surgery done within 3 months. If the ophthalmic specialist nurse detects any complications or the IOP was equal or above 30 mmHg, then the patient would be seen at 1 day, 7 days, and 1 month postsurgery for both eyes. A single dose of 500 mg oral acetazolamide would be administered for those with IOP above 30 mmHg. 30 mmHg was chosen as a cutoff as previous studies have showed that at this level, there is a significant increase in optic disc cupping [10]. Complications include wound leak, IOP higher or equal to 30 mmHg, peaked pupil, IOL dislocation, and severe inflammation.

All surgeries were performed at a day surgical centre (Wu Ho Loo Ning Cataract Centre, Tseung Kwan O Hospital). Cases were performed under either topical anaesthesia or retrobulbar anaesthesia, depending on the patient's cooperation and/or surgeon's preference. Standard phacoemulsification with clear corneal incision was performed (Infiniti vision system, Alcon Inc., Fort Worth, USA). Intracameral injection of cefuroxime (1 mg in 0.1 mL) was performed routinely unless there was any known related allergy. Standard postoperative regimen of 0.5% chloramphenicol with 0.1% dexamethasone eye drops was prescribed. No routine prophylactic medications were prescribed for IOP lowering.

Primary outcome measures include visual improvement postsurgery and patient satisfaction via a questionnaire. The questionnaire consists of 3 sections. The first and second section is completed 1 month after the first and second cataract surgery, respectively. Subjects are asked to rate their overall experience, level of reassurance, and explanation received during follow-up, and confidence level towards the follow-up health care professional on a scale of 1–5, with 1 representing very satisfied and 5 representing very dissatisfied. In the third section, subjects are asked to state their preference towards NDR versus NNDR and also the venue of follow-up. Other outcome measures include preoperative visual acuity, cataract characteristics, surgical details, surgeon ranking, phacoemulsification energy, and complications. Visual acuity was measured with the use of pinhole and Snellen chart. A small pupil was defined as less than 5 mm after pupil dilatation. High myope was defined as myopia of 6 diopters or above. Dense cataract was defined as nuclear sclerosis of grade 3 or above.

All questionnaires were completed on day 7 after surgery for both eyes, evaluating the patient satisfaction level for the NDR group and NNDR group, at a scale from 1 to 13, with 1 presenting very satisfied and 13 presenting very unsatisfied. At day 7 after surgery for the second eye, the patient will be asked to state his preference in the following areas: (1) location—review at the Day Surgery Center versus the outpatient clinic, and (2) schedule—the NDR versus NNDR schedule.

Paired *t*-test was used for parametric data analysis, Wilcoxon signed rank test for nonparametric data, and Chi-square test for categorical data. Means were expressed as mean ± standard deviation (SD). For comparison of visual acuity between NDR and NNDR, Snellen visual acuity was converted into logMAR visual acuity to obtain geometric progression. Statistical significance was defined as *p* < 0.05. Analysis was conducted with SPSS version 20.

## 3. Results

**Figure 2:**
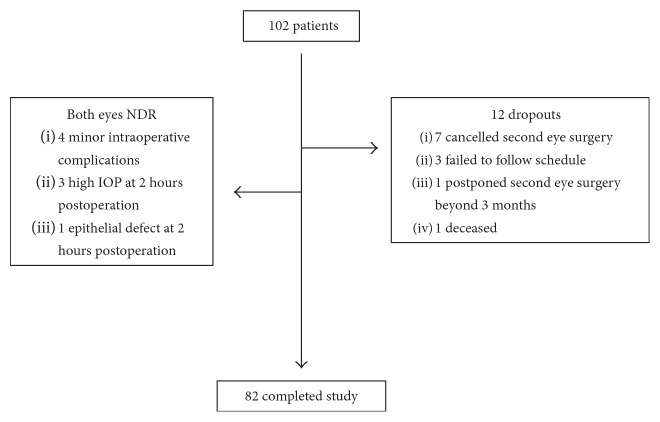
The recruitment outline.

A total of 102 patients were recruited with the recruitment outline shown in Figure 2. Among them, there were 12 dropouts: 7 patients cancelled their second eye surgery, 3 patients failed to follow the study schedule, 1 patient had his second eye surgery postponed beyond 3 months, and 1 patient passed away after the first eye surgery. Eight patients had their NNDR changed to NDR group for various reasons. This includes 4 cases that encountered minor intraoperative complications: 2 had wound burn, 1 had repeated intraoperative iris prolapse, and 1 required monitored anaesthetic care due to poor cooperation. The remaining 4 were found to have minor complications at 2 hours postsurgery review by a nurse specialist (ML): 3 had high IOP (range 35–41 mmHg) and were given a single dose of oral acetazolamide (500 mg) and 1 had a corneal epithelial defect.

Data from the remaining 82 patients were available for analysis; the mean age was 74.9 ± 7.8. Forty-eight were female and thirty-four were male. The operative details are shown in Table 1. For the surgery, the case mix of surgeries and surgeons were representative of our norm. The mean preoperative visual acuity of the NDR group and NNDR group were similar (paired *t*-test, *p* = 0.84). Difficulty and density of the cataract in the NDR and NNDR group were noted preoperatively by noting down characteristics including shallow anterior chamber, small pupil, sunken globe, high myope, dense cataract, and posterior subcapsular cataract. These parameters were comparable among the two groups. Density of the cataract was also reflected by recording the total dissipated phacoemulsification energy postoperatively. The cumulated dissipated energy (%-s) required in the NDR group was 16.11 ± 10.24 and that in the NNDR group was 14.86 ± 9.02, with no statistically significant difference (paired *t*-test, *p* = 0.38). The surgical details and rank of surgeons were also comparable between both groups.

There was no statistically significant difference in the visual acuity between NDR and NNDR at baseline and at 7 days or 1 month after phacoemulsification (see Table 2). The preoperative visual acuity for the NDR and NNDR groups was 0.3 +/− 0.1 and 0.3 +/− 0.2, respectively (*p* = 0.6). The visual acuity at day 7 postoperation for the NDR and NNDR groups was 0.5 +/− 0.2 and 0.5 +/− 0.2, respectively (*p* = 0.2). The visual acuity at 1 month postoperation for the NDR and NNDR groups was 0.5 +/− 0.2 and 0.5 +/− 0.2, respectively (*p* = 0.7). No complications such as wound leaks, peaked pupil, IOL dislocation, or severe inflammation were noted in either group at D0, D1, or D7. Three patients had IOP higher than 30 mmHg noted at D0 as mentioned above and were given one dose of 500 mg oral acetazolamide and reviewed the next day as per study protocol. No subjects were noted to have IOP higher than 30 mmHg at other study time points. For the NDR group, IOP at D0 and D7 were 22.2 +/− 5.4 and 11.2 +/− 3.9 mmHg, respectively. For the NNDR group, IOP at D0 and D7 were 22.2 +/− 5.6 and 11.9 +/− 3.4 mmHg, respectively. There was no statistical significance of D7 IOP between the NDR and NNDR groups (paired *t*-test, *p* = 0.3).

There was no significant difference in patient satisfaction level between the NDR group and NNDR group (5.33 ± 1.1 versus 5.09 ± 1.29, *p* = 0.06, Wilcoxon signed rank test). For patient preference comparing NDR schedule versus NNDR schedule, the vast majority showed no preference (65%). 22% preferred the NNDR schedule while 13% preferred the NDR schedule. For patient preference comparing review at the Day Surgery Center versus the outpatient clinic, the vast majority were neutral (82%), while 13% preferred the Day Surgery Center.

## 4. Discussion

A number of studies with various designs have been conducted to study the necessity, indications, and optimal timing of the first postphacoemulsification review.

A summary of studies focusing on NDR is shown in Table 3 [11–14]. The majority of the NDR were conducted by doctors. In studies where NDR were deemed necessary, the main purpose is to provide management for high IOP. As a high incidence of complications were found on NDR in complicated phacoemulsifications, NDR were deemed necessary in this group of patients as well.

A summary of prospective studies looking into the optimal timing of the first postphacoemulsification review and their outcome is shown in Table 4 [5, 15–19]. The majority of the NDR were conducted by doctors, while in one study, SDR was conducted by a nurse with a pen torch. Although the patients were reviewed at different time points in different studies, a few common results can be observed as follows: (1) An early SDR is more effective in picking up postoperative IOP spikes, and (2) in the long run, the visual outcome is comparable irrespective of whether the review was conducted on the same day, next day, or even later. However, although many of these studies conclude that SDR is safe and efficacious when compared to NDR, their follow-up protocol and logistics fail to ensure patient safety, as complications such as painless iris prolapse and corneal abrasions have been missed in the SDR group.

The results of the present study is consistent with the above conclusion in that SDR is comparable to NDR in both efficacy and safety. SDR is advantageous in that its timeframe allows more efficacious detection in IOP spikes. This is of even greater significance to glaucoma patients, who have a higher tendency to develop deleterious IOP spikes. Ahmed et al. found that at 3–7 hours postoperation, 18% of nonglaucoma patients and 46% of glaucoma patients were found to have IOP > 28 mmHg, while 4% of nonglaucoma patients and 19% of glaucoma patients had IOP > 40 mmHg [7]. We took extra precautions to enhance safety for SDR patients; only uneventful phacoemulsification was included in the present study, and all cases with IOP spikes were reviewed the next day. Our SDR was conducted by an ophthalmic nurse who had undergone accredited slit lamp training. As such, we had no complications that had gone undetected in either group. SDR is a more convenient and economical arrangement, especially for patients with mobility issues or who live far away from the hospital.

One concern for implementing SDR would be the issue of detecting postcataract endophthalmitis. The incidence of postcataract endophthalmitis has dropped since the introduction of intracameral cefuroxime prophylaxis. Prior to its introduction, the incidence ranges from 0.3% to 1.2% [20–23]. With the introduction of intracameral cefuroxime, the risk is further reduced by fivefold, ranging from 0.014% to 0.08% [24]. The above range refers to all cataract surgeries and encompasses both the uneventful and complicated. As the ESCRS study group found a fivefold increased endophthalmitis risk in the presence of surgical complications, the incidence of endophthalmitis following uneventful phacoemulsification is even lower [25]. The mean time between cataract surgery and diagnosis of endophthalmitis has been quoted to range from 6 to 13 days [15, 17]. The efficacy of NDR in detecting endophthalmitis is, therefore, extremely low. Although there has been a case report of endophthalmitis detected at day 1 postsurgery, the patient had underlying risk factors including an anaesthetic cornea from history of herpes zoster ophthalmicus and history of infective keratitis [26]. In this new era, we believe that the main role for the first postphacoemulsification review lies in detecting IOP spikes. We therefore advocate replacing NDR with SDR, rather than delaying the first review till late. The second review, which can be scheduled at 5 to 7 days postsurgery, would be much more effective in detecting endophthalmitis.

Implementation of SDR, shared care with a nonophthalmologist, and patient review at the Day Surgery Center has allowed us to streamline our review protocol, allocate our resources more effectively, and facilitate the development of high volume cataract surgery in all aspects, while maintaining health care standard and patient safety at the same time. Although our study included surgical cases conducted under local anaesthesia only, we believe that surgery under general anaesthesia can also be safely included. Shared care with nonophthalmologists is feasible provided that appropriate training has been undertaken and appropriate equipment are used. Lastly, the importance of perioperative patient education in postoperative care and warning symptoms cannot be overemphasized.

Our study is unique in several aspects. The study is conducted among an Asian population and background. A randomized crossover design has been adopted to allow better comparison. We also take patient satisfaction into consideration. To the best of our knowledge, this is the first postphacoemulsification review study of such nature conducted among Asian subjects. Like many metropolitan cities worldwide, Hong Kong's public transport and travel are known to be particularly convenient. In general, our patients prefer SDR to NDR, even when transportation is already less of an issue. Limitations of our study include a relatively small sample size and not adopting validated patient satisfaction questionnaires. Also, examiners may be able to distinguish NDR patients despite NDR and NNDR blinding.

In conclusion, the present study showed that NNDR combined with SDR by a nurse specialist is efficacious and safe in uneventful phacoemulsification in the era of intracameral cefuroxime. Patient satisfaction did not show any preference between NDR and NNDR and further supports NNDR with the use of SDR. These findings bear significance and may influence the current practice of NDR common in Asian countries.

## Figures and Tables

**Table 1:** Table showing cataract difficulty, intraoperative details, and parameters.

	NDR	NNDR	*p* value
	Mean ± SD or number (%)	Mean ± SD or number (%)	
Preoperative VA	0.3 ± 0.13	0.3 ± 0.16	0.84^∗^

*Difficult cataract*			
Shallow AC	5 (6.1)	6 (7.3)	0.76^#^
Small pupil	16 (19.5)	19 (23.2)	0.57^#^
Sunken globe	17 (20.7)	17 (20.7)	1.0^#^
High myope	6 (7.3)	6 (7.3)	1.0^#^
Dense cataract	7 (8.5)	10 (12.2)	0.44^#^
PSC cataract	9 (11)	14 (17)	0.27^#^

*Operation details*			
Wound site			
Temporal	72 (87.8)	71 (86.6)	0.82^#^
Superior	10 (12.2)	11 (13.4)

*Anaesthesia*			
TA	77 (93.9)	76 (92.7)	0.76^#^
RA	5 (6.1)	6 (7.3%)

*Surgeon rank*			
T	8 (9.8)	10 (12.2)	0.62^#^
S	74 (90.2)	72 (87.8)	

Require suture	5 (6.1)	3 (3.7)	0.47^#^

Phaco CDE (%-sec)	16.11 ± 10.24	14.86 ± 9.02	0.38^∗^

VA: visual acuity; AC: anterior chamber; PSC: posterior subcapsular cataract; TA: topical anaesthesia; RA: regional anaesthesia; T: trainee; S: specialist; Phaco CDE: phacoemulsification cumulative dissipated energy.

^∗^Paired *t*-test, ^#^Chi-square test.

**Table 2:** Table showing visual acuity and intraocular pressure at different study time points.

	NDR	NNDR
Baseline VA^∗^	0.31 +/− 0.14	0.3 +/− 0.16 (*p* = 0.6)^#^
D7 VA^∗^	0.49 +/− 0.17	0.53 +/− 0.15 (*p* = 0.2)^#^
M1 VA^∗^	0.49 +/− 0.17	0.52 +/− 0.16 (*p* = 0.7)^#^
D0 IOP	22.2 +/− 5.4	22.2 +/− 5.6 (*p* = 0.9)
D7 IOP	11.2 +/− 3.9	11.9 +/− 3.4 (*p* = 0.3)

NDR: next day review; NNDR: no next day review; VA: visual acuity; D7: day 7 postoperation; M1: month 1 postoperation; D0: day 0 postoperation.

^∗^Presented in Snellen VA; ^#^calculated by converting Snellen into logMAR VA.

**Table 3:** Table summarizing studies on postphacoemulsification next day reviews.

Study/year	Pt (*n*)	Design	Patient examined by	Results	Conclusion
Dinakaran/2000	71	Retrospective review of NDR case notes	Doctor	10% high IOP 30% corneal edema	NDR necessary to manage IOP rise
Herbert/1999	392	Retrospective review of NDR case notes	Nurse	2% high IOP 0.2% painless iris prolapse	NDR necessary to manage complications
Tan/2000	238	Prospective analysis of NDR	Doctor	Uneventful phaco: 4% corneal edema 1% high IOP Complicated phaco: 71% complications	NDR not necessary for uneventful surgery
Whitefield/1995	100	Prospective analysis of NDR	Doctor	3% had IOP >30 mmHg	Need for NDR questionable and probably unnecessary

NDR: next day review; phaco: phacoemulsification.

**Table 4:** Table summarizing prospective comparison studies looking into the optimal timing of the first postphacoemulsification review.

Study/year	Pt (*n*)	Design	Patient examined by	Results	Conclusion
Chatziralli/2012	291	Prospective RCT review at: NDR or W2	Doctor	VA comparable at D28 Complications: nonscheduled consults 3 in NDR group, 2 in W2 group	NDR can be omitted
Saeed/2007	233	Prospective RCT review at: 2 hrs or W2	Doctor	Significantly more IOP spikes detected at 2 hrs At 2 weeks, VA and Cpx similar	Defer review to 2 weeks is safe, provided that transient IOP spike not deemed clinically deleterious
Tranos/2003	141	Prospective cohort review at: 4–6 hrs or NDR	Doctor	Significantly more IOP spikes detected at 4–6 hrs At 3 weeks, VA and Cpx similar	SDR safe and more efficacious than NDR
Tinley/2003	174	Prospective RCT review at: SDR or NDR	SDR by nurse NDR by doctor	At 2 weeks, VA and vision-related QOL similar Complications: 2 cases of iris prolapse in SDR group	SDR is as safe and efficacious as NDR
Tufail/1995	387	Prospective cohort review at: 4 hrs or NDR	Doctor	At 1 week, VA is similar Complications: 1 case of iris prolapse in each group	SDR is as safe and efficacious as NDR

RCT: randomized controlled trial; NDR: next day review; SDR: same day review; W: week; VA: visual acuity; hrs: hours; Cpx: complications.

## Additional Points

*What Was Known*. The practice of NDR is not evidence based. *What This Paper Adds*. To explore the optimal timing of first postphacoemulsification review while assuring patient safety; a randomized crossover study design to allow even fairer comparison; to compare SDR with NDR in terms of safety, efficacy, and patient satisfaction in metropolitan Hong Kong, where the culture and traffic infrastructure differ largely from the West; and incorporate nurse-led SDR to streamline cataract surgery efficiency.
